# Understanding internal migration in the UK before and during the COVID-19 pandemic using twitter data

**DOI:** 10.1007/s44212-022-00018-w

**Published:** 2022-11-29

**Authors:** Yikang Wang, Chen Zhong, Qili Gao, Carmen Cabrera-Arnau

**Affiliations:** 1 Centre for Advanced Spatial Analysis, University College London, London, UK; 2 Department of Geography and Planning, University of Liverpool, Liverpool, UK

**Keywords:** Migration, Human mobility, Geocoding, Twitter, COVID-19

## Abstract

The COVID-19 pandemic has greatly affected internal migration patterns and may last beyond the pandemic. It raises the need to monitor the migration in an economical, effective and timely way. Benefitting from the advancement of geolocation data collection techniques, we used near real-time and fine-grained Twitter data to monitor migration patterns during the COVID-19 pandemic, dated from January 2019 to December 2021. Based on geocoding and estimating home locations, we proposed five indices depicting migration patterns, which are demonstrated by applying an empirical study at national and local authority scales to the UK. Our findings point to complex social processes unfolding differently over space and time. In particular, the pandemic and lockdown policies significantly reduced the rate of migration. Furthermore, we found a trend of people moving out of large cities to the nearby rural areas, and also conjunctive cities if there is one, before and during the peak of the pandemic. The trend of moving to rural areas became more significant in 2020 and most people who moved out had not returned by the end of 2021, although large cities recovered more quickly than other regions. Our results of monthly migration matrixes are validated to be consistent with official migration flow data released by the Office for National Statistics, but have finer temporal granularity and can be updated more frequently. This study demonstrates that Twitter data is highly valuable for migration trend analysis despite the biases in population representation.

## Introduction

The COVID-19 pandemic has greatly impacted people’s location choices (Batty, 2020). National and regional lockdowns, economic depression, and working from home policies have significantly decreased short-term human mobility such as daily trips and tourism, and have also affected people’s home location choices – specifically, residents have been moving out of large cities due to the pandemic (Haslag & Weagley, 2021; Willberg et al., 2021). This effect may not be limited to the pandemic period and could last forever (Batty, 2022). The study of migration is the component of understanding population change and the social problems that follow (Clark, 1985). The accurate and timely measurement of changes in internal migration during a pandemic is essential for quantitatively assessing the impact and effectiveness of restrictive policies and to help develop recovery strategies for the post-pandemic phase (Martin & Bergmann, 2021).

Considering the rapid changes of the pandemic, including disease transmission, virus mutation and changing policies, an effective migration monitoring method should meet the following three requirements of fine temporal granularity, frequent updating, and affordable costs in data aquation and computing. Traditional migration monitoring methods mainly rely on governmental official data (Abel & Sander, 2014; Bell et al., 2015), which are retrospective and offer coarse temporal granularity (i.e., poor temporal resolution due to the delay of data release and the low updating frequency). Internal migration studies (ONS, 2021b) normally have a temporal granularity of 1 year, whereas international analyses (UNDP, 2013) have a granularity of 5 to 10 years and suffer from years of delay in releasing the result.

The emerging big data sources capturing where people are have presented a high potential for tracking mobility patterns (Li et al., 2021). For example, information technology (IT) companies, such as Google, Apple, Facebook, Tencent and Baidu, publish near real-time mobility indices during the pandemic. However, these indices have low spatial granularity (usually on a city scale) and only include movements within and between large cities or large administrative regions (provinces or states), ignoring migration between cities and rural areas. In addition, previous studies (Huang et al., 2021; Terroso-Saenz et al., 2022; Zhang & Cheng, 2022; Zhong et al., 2022) have mainly focused on short-term trips, such as daily mobility or tourism, whereas long-term migrations, i.e., home relocation, have not received scant attention. Mobile phone data is another important source of tracking migration but is very expensive and difficult to access (Bonnetain et al., 2021). Therefore, there is a lack of an effective monitoring tool for migration in the context of important social events such as COVID-19. In contrast, social media data, such as Twitter has the advantages of real-time updates, free access, and fine spatial and temporal granularity. It offers us the opportunities to fulfil the needs of capturing internal migration patterns across the whole country, including both inter/intra-city migration and city-rural migration.

This paper aims to present the utility of Twitter data for fine-grained and economical monitoring of migration in the context of COVID-19. We present the application through a case study of internal migration in the United Kingdom (UK) before and during the COVID-19 epidemic. Through geocoding of Twitter place attributes and estimation of users’ home locations, we obtain monthly origin-destination (OD) matrices of internal migration in the UK and propose five indices to describe migration patterns. Our approach is adaptable to monitoring internal and international migration in other social events.

The rest of the paper is organised as follows. Section 2 reviews previous literature related to migration studies using various kinds of data sources. The data sources used in this research are described in Section 3. Section 4 is dedicated to the data-driven methodology for geocoding and detecting instances of migration, including the set of migration indicators used in subsequent sections of the paper. In Section 5, we introduce the case study concerning migration in the UK before and during the pandemic. Results and limitations are discussed and concluded in Section 6.

## Literature review

Migration has always been an important topic in the urban geography domain. Quantifying internal and international migration is the basis of further migration studies, such as analysing spatial and temporal patterns (Davis et al., 2013), underlying causes (Parrish et al., 2020), and social impacts of migration (Fagiolo & Mastrorillo, 2014). Previous studies in quantifying migration mainly rely on official statistical data (e.g., census and survey data). For instance, the UK Office for National Statistics (ONS) provides annual Local Authority District (LAD)-scale internal migration analyses using census, survey, and administrative registers data (ONS, 2021c). The result has a yearly temporal granularity, and the release has a year delay (i.e., the mid-2019 to mid-2020 result is released in mid-2021). Similarly, the Internal Migration Around the GlobE (IMAGE) project analyses internal migration in 193 United Nations member States using official data (Bell et al., 2015). International migration flows based on government official data are usually estimated from sequential stock tables since there is a lack of official statistics in most countries except those belonging to the European Union. Consequently, data related to international migration tends to have a coarse temporal granularity, such as 5 or 10 years (Abel & Sander, 2014). Due to the delays in releasing data, the migration analysis using official data is usually retrospective. Given the rapid evolution of the COVID-19 pandemic, retrospective migration monitoring using official data with years of delay cannot meet the needs.

In addition to official statistics (Fielding & Ishikawa, 2021; ONS, 2021b), multiple emerging data sources have been explored for migration analysis including public transport data, mobile phone data (Kang et al., 2020; Pullano et al., 2020) and social media data (Huang et al., 2020; Terroso-Saenz et al., 2022). Public transport data is useful for capturing intra-city mobility but is hard to track inter-city and city-rural migrations. Mobile phone location data has finer spatial and temporal granularity but is expensive to obtain and process (Bonnetain et al., 2021; Willberg et al., 2021). Social media data has the advantages of being updated in real-time, actively collected and freely accessed. It has been demonstrated to be valuable for monitoring human mobility in (near) real-time during the COVID-19 pandemic in a way that preserves the privacy of users (Gao et al., 2019; Hu et al., 2021; Huang et al., 2020; Li et al., 2021; Sîrbu et al., 2021).

In particular, Twitter has become the most used social media platform to source data since it grants free and open access to about 1% of its content through the official Application Programming Interface (API) (Martín et al., 2020). As a result, thousands of studies leverage Twitter data every year (Karami et al., 2020) for a wide range of purposes, such as spatial analysis (Bao et al., 2017; Lai, 2019), demographics (Longley & Adnan, 2016), and epidemic surveillance (Shin et al., 2016). Zagheni et al. (2014) extract migration patterns by analysing the location where tweets were posted, while Moise et al. (2016) estimate the origin of immigrants by the language in tweets. In any case, Twitter data has been proven useful for activity location estimation (Steiger et al., 2015), monitoring flows between regions (Blanford et al., 2015), and depicting urban boundaries (Yin et al., 2017).

Twitter data has been widely used in COVID-19-related research due to its public accessibility, high population coverage, and real-time updates. For example, Twitter data has been used to analyse public sentiment and perceptions on the COVID-19 pandemic (Boon-Itt & Skunkan, 2020) and vaccination (Ali et al., 2021) to describe and predict the COVID-19 spread and outbreaks (Jahanbin & Rahmanian, 2020), to evaluate the impact of COVID-19 on ride-hailing services (Morshed et al., 2021), and to analyse the creation and prevalence of stigma by referring to the COVID-19 pandemic as *Chinese Virus* (Budhwani & Sun, 2020). With regards to human mobility during the COVID-19 pandemic, previous studies have used Twitter data to assess the impact of restriction policies on short-term trips (Huang et al., 2020; Huang et al., 2021) and to analyse and predict how population movements may affect disease transmission (Bisanzio et al., 2020). Terroso-Saenz et al. (2022) used geo-tagged Twitter data to analyse internal mobility in Spain during the first wave of COVID-19, demonstrating that Twitter is a reliable source for capturing mobility trends.

Despite extensive studies during the COVID-19 pandemic using Twitter data, very few studies have been conducted for migration monitoring that meets all the three above-mentioned requirements – fine-grained, near real-time and economical. Drawing on these gaps, this study uses Twitter data to evaluate internal migrations in the UK before and during the COVID-19 pandemic. Moreover, we propose multiple indicators to reveal migration patterns comprehensively.

## Study material

### Twitter data

This study is based on an anonymised dataset of 182 million tweets that were sent in the UK from the beginning of 2019 to the end of 2022 and were collected using the Twitter Developer API. Each tweet in the dataset contains a list of attributes: tweet ID, user ID, time, tweet content, Internet Protocol (IP) address and estimated place; these are detailed in Table 1. The place attribute was generated according to the geotag of the tweet if applicable (when users decide to assign a place to the tweet, they are presented with a list of candidate places), or was estimated from the user’s IP address. Therefore, the tweet place indicates a place associated with the tweet but not necessarily the place where it originated.

**Table 1:** Example of tweet data fields and values

Data Name	Data Type	Example
Tweet ID	Integer	123,456,789,012,345,678
User ID	Integer	123,456,789
Tweet Content	String	What a lovely day!
Time	String	Fri Dec 31 09:00:01 + 0000 2021
Place	String	Hyde Park
IP Address	String	100.0.0.0

### UK geographical data

The spatial unit of this study is LAD. The UK government has LAD-scale census and population movement data to facilitate our research. LADs are updated annually by the UK government. Here, we utilised the latest version published in April 2021 from ONS (2021a), which contained 374 LADs.

Although each tweet had a place attribute, places were not derived from an official definition; furthermore, the spatial scale of the place attribute was inconsistent, ranging from Points of Interest (POIs) or addresses, such as a park or a restaurant, to regional or country-wide scales, such as Wales. In order to homogenise the variety of spatial scales represented by tweet places, we matched these places to LADs, which constitute one of the official subnational territorial divisions in the UK and are considered the most suitable administrative division for our analysis of migration patterns.

### COVID-19 data

COVID-19 data was sourced from the UK government (Public Health England, 2022), including daily new cases of COVID-19 and the vaccination rate of the first dose in the UK since the beginning of the pandemic. Daily new cases were smoothed by a seven-day rolling average to reduce the impact of incorrect reporting and misreporting. Our interest was in analysing the relationship between this data and data representing migration movements.

## Research methodology

We achieved migration monitoring through a three-step data-driven method. First, the place attribute associated with each tweet was matched to the corresponding LAD by geocoding. Next, we estimated each user’s home location using tweet place and then quantified migration by comparing the user’s home address in two non-overlapping time periods. Finally, we defined five indicators to explore migration and analyse migration patterns and assessed their correlation with population density using linear regression to assess the variation in impacts on cities of different sizes.

### Tweet place geocoding

First, tweets whose place reported at larger than LAD spatial scale were removed from the dataset in order to avoid ambiguity. Out of the total collected tweets, 161 million (89%) had valid place attributes at or below the LAD scale. We then constructed a lookup table matching lower-level geographic hierarchies to LADs by place names, which could assign 3.3% of places to LADs. For the remaining unmatched tweets, we used Bing Maps Geocoding API to obtain the corresponding addresses, administrative districts and bounding boxes of the places of these tweets. 52.6% of places were assigned to LADs.

If a returned administrative division was not included in the LAD list and lookup table, we carried out ‘intersection geocoding’ that computes the intersection index of its bounding box according to Bing Maps Geocoding API and each LAD as follows:

$$I\left( Place, LAD\right)=\frac{Area\ of\ intersection\ with\ LAD}{Area\ of\ the\ place\ bounding\ box},$$

where place is the place attribute of the tweet. If *I*(*Place*, *LAD*) > 65%, then the corresponding tweet was considered to belong to that LAD.

After applying the above procedures, approximately 160 million tweets were successfully geocoded at the LAD scale. On average, 235 thousand users'  home locations per month were successfully detected at the LAD scale.

### Detecting migrations

Human trajectories are characterised by a high degree of temporal and spatial regularity. Individuals tend to return to a small set of locations, such as their homes and workplaces (González et al., 2008). Given this observation, we assumed that the home location of each user corresponds to the LAD, where they posted the most in a specified period. In our case, we assumed that home location can be identified when the number of LAD-geocoded tweets was greater than 2 and at least 65% were from the same LAD in a month. We considered the above assumption a robust way for home LAD estimation.

We defined that a user has migrated when the home locations corresponding to two non-overlapping periods were different. In particular, assuming that migratory movements happened more frequently during COVID-19 because of safety concerns, finances or other considerations (Haslag & Weagley, 2021), the OD matrices for the case study presented in later sections were generated by comparing home locations between two consecutive months. To validate the migration flows detected from Twitter data, we also generated an annual migration OD matrix and compared it with ONS data.

### Indicators of migration patterns

Based on the identified migration matrix, spatial and temporal trends of migration can be evaluated. Considering the changes in people’s choice to enter or leave cities during the pandemic and the attractiveness of each area, we defined the following indicators to evaluate the migration patterns and heterogeneity before and during the COVID-19 pandemic:

- **Migration rate**: the number of migrations divided by the total number of Twitter users whose home locations were successfully detected in both months.
- **City migration index**: the number of migrations in/out of LADs belonging to large cities divided by the total number of migrations. LADs were attributed to large cities on the basis of population density.
- **Net migration index**: the net number of migrations (inflows minus outflows) for a LAD divided by its census population.
- **Migration share**: the number of migrations moving from a selected city to each LAD, divided by the total number of migrations from that city.
- **Recovery index**: the total number of migrations (inflows plus outflows) for a LAD in 2021 divided by the corresponding number in 2020.

Relative values rather than absolute values were used to overcome data bias, with the assumption that the level of bias remained the same before and during the COVID-19 pandemic. To evaluate the patterns and variability of people’s choice of location and the relationship between city size and migration activity, a correlation analysis of the net migration index and (log) population density in each year is presented. We also conducted the linear regression between recovery index and (log) population density to examine the differences in post-pandemic recovery between urban and rural areas.

## Results and discussion

### Validation of detected migration flows

To assess the validity of the migration matrices generated from Twitter data, we compared the yearly matrix with annual internal migration data from ONS (2021b) in England and Wales in the year ending in June 2020. The ONS data contains flows of migrants between each pair of LADs in England and Wales, and can be taken as the ground truth.

**Fig. 1:**
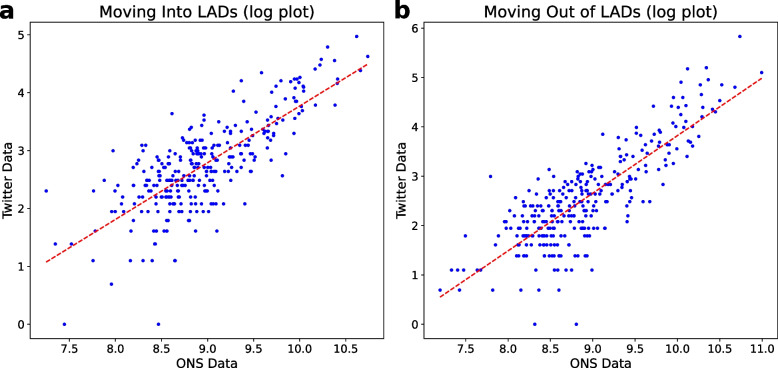
Comparison plot of LAD migration numbers as determined from Twitter data (localised Twitter users > 50 in England and Wales in the year ending June 2020) with corresponding ONS internal migration data: (**a**) moving into LADs, (**b**) moving out of LADs

The City of London was considered an outlier and removed from the list of LADs due to the fact that in this LAD, there are few residents but a high number of people who travel there daily for work, so this can lead to the misidentification of home locations. We also removed LADs with no more than 50 located Twitter users for the purpose of this validation since too few of users would lead to the migration being incorrectly identified as 0. Figure 1 depicts the linear regression of the Twitter-based estimation of the yearly migration matrix between June 2019 and June 2020 and the corresponding migration data from ONS. The R-value for moving in and out of LADs were 0.77 (*p*-value: 1e-61) and 0.82 (p-value: 2e-77), respectively, validating the effectiveness of the method.

### Trend analysis

#### Migration before and during the COVID-19 pandemic

**Fig. 2:**
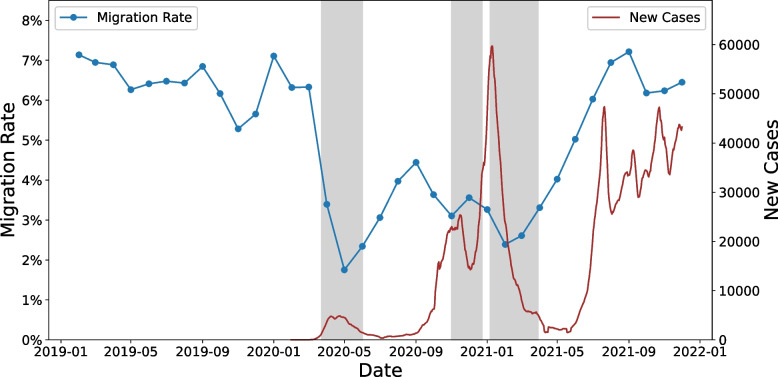
Monthly migration rate and new COVID-19 cases in the UK, 2019-2021

This study first monitored the movements and detects abnormal patterns and behaviours at small temporal scales. Figure 2 illustrates migration rates for every pair of consecutive months in the time period spanning from 2019 to 2021, with the date marked on the first day of the second month. As different regions in the UK varied in their COVID-19 restrictions, we have marked the periods during which most parts of the UK went into lockdown as grey in the figure.

The migration rate exhibited a strong association with both the evolution of the COVID-19 pandemic and government restrictions. In 2019, the average monthly migration rate was 6.41%, whereas the average rates in 2020 and 2021 were 3.81% and 5.13%, respectively. The first lockdown was introduced on March 23rd, 2020, and then steadily eased across the UK during late May and June 2020; correspondingly, the migration rate fell sharply from 6.33% to 3.39% in March-April and achieved a low of 1.75% in April-May. COVID-19 cases began to increase again in early October 2020 and the second lockdown was introduced at the end of that month, resulting in a drop in the migration rate to 3.1% in October-November. Then, following a partial easing of restrictions for Christmas, the UK went into a third lockdown and the migration rate dropped again in January-February 2021, to 3.26%. Finally, after the three rounds of lockdown, the migration rate gradually rose to pre-pandemic levels in Autumn 2021. Although the number of new cases of COVID-19 increased and remained high from summer 2021, the migration rate was not affected.

The decrease in the number of migrations might be attributed to the pandemic and restriction policies – such as stay-at-home rules, prohibitions on leaving restricted areas, working from home policies and public events cancellations. For example, the curfew (do not stay out of home overnight) and regional lockdowns (do not leave Tier-4 restricted areas) announced by the UK government effectively banned most internal migrations. Our result is consistent with the trend revealed in ONS research, which found that one of the most notable impacts of the pandemic on internal migration was the 11.5% reduction relative to the previous year in the number of moves in the year ending mid-2020, which occurred because of the first national lockdown restricting people from moving homes (ONS, 2021c). Notably, the value of migration index did not decline in response to the increased number of daily new cases in the summer of 2021 onwards due to the rapid spread of the delta variant of COVID-19, most likely due to mass vaccinations and the absence of lockdowns. In a study of migration in the US that relied on mobile phone data, Kishore et al. (2021) noted that internal migration increased in the period after restriction policies were announced and before their implementation; such a phenomenon was not evident in our monthly results, as only 3 days passed between the announcement of the first lockdown on March 23, 2020, and those measures legally coming into force on March 26.

**Fig. 3:**
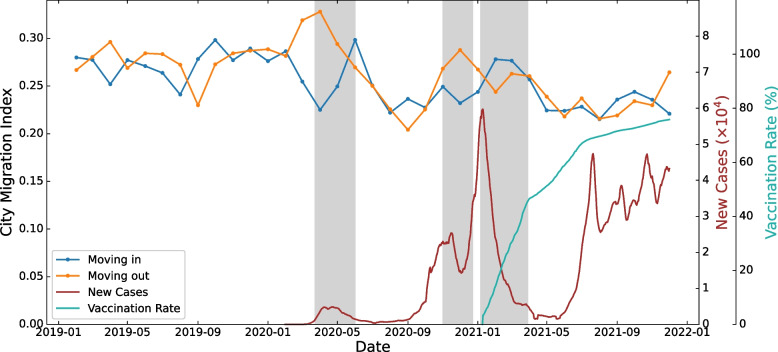
Monthly city migration indices for moving into and out of large cities in the UK, 2019-2021

To explore trends in people moving in and out of large cities during the pandemic, we first defined the LADs whose population density was in the top 10% as belonging to large cities. Then, we determined the city migration indices of people moving into and out of those LADs, and calculated for every pair of consecutive months. The results are shown in Fig. 3, with lockdown periods marked in grey as well. The vaccination rate of the first dose is shown in turquoise.

The city migration indices also showed a strong correlation with pandemic measures. The two largest gaps between moving in and out of large cities were in April and December 2020, during the first and second lockdown, respectively, meaning that residents of large cities were exiting those areas. When the pandemic subsided, the migration index of moving into large cities surpassed the moving-out index, possibly because a small proportion of people were returning to the cities, with most choosing to stay out of cities until the end of 2021. During the third lockdown from January to March 2021, the trend was different from the previous two lockdowns: fewer people moved out of the cities as they moved in. The increase in vaccination rate and gradual decline in daily new cases possibly eased people’s willingness to move out, although new cases were at a record high at the beginning. Another possible reason is that some people who moved out of cities before and during Christmas were returning after the holidays. To draw a firm conclusion, we need to extend the study for longer-term monitoring. Nevertheless, the unbalance between in and out flows consistently exists. In particular, the amount of net moving in after the pandemic is far less than that of moving out during the lockdowns. Over the other periods, the moving in and out indices were mostly in similar fluctuations, indicating no significant trend of moving into or out of cities. These phenomena persisted when the population density threshold for classifying large city LADs was changed from 10% to any value between 5% and 20%. On the other hand, the migration index of large cities is about 0.2~0.3 which implies an index of 0.7~0.8 for small cities and rural areas. This reveals a constant tendency to move in and out of these areas, both before and during the pandemic.

### Where to move before and during the COVID-19 pandemic?

**Fig. 4:**
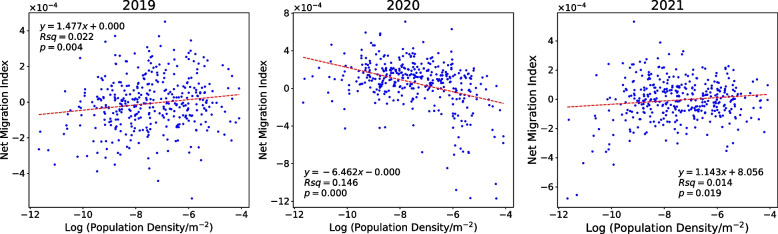
The relationship between net migration index and (log) population density in 2019, 2020 and 2021

To further explore the population losses of large cities during COVID-19, we examined the relationship between the net migration index and (log) population density in 2019, 2020 and 2021 (Fig. 4). In addition to the LAD of the City of London, we removed North Northamptonshire and South Northamptonshire from this analysis due to a lack of census data, and also the Isles of Scilly due to their very low population (2200 people). In 2019 and 2021, the R-square values were less than 0.022, with positive correlation coefficients of 1.477 and 1.143, indicating the net migration index does not depend on population density (city size), thus no occurrence of city shrinking or urban depopulation. This finding presents a relatively stable and slow urbanisation trend whereby people migrate to large cities in the UK, consistent with previous (ONS, 2021d). But the pattern changed because of the shock of the pandemic. The 2020 data yielded a substantially higher R-square value of 0.15 and a regression coefficient of − 6.46, implying the pandemic hit the large cities the hardest. In addition, given the negative net migration index, larger cities show a higher rate of outflow to other regions. One of the potential reasons is the higher risk of the pandemic in large cities because of the high population densities and movement (Jamal et al., 2022). These findings inform the government to pay more attention to large cities to timely control the potential population loss caused by pandemic risks. While such population loss ceased in 2021, it does not show a significant trend of moving into cities. As working arrangements in the post-pandemic period were often hybrid (e.g. going to the office a few days a week), commuting levels were lower than that in the pre-pandemic period, making the suburbs relatively more popular (Ramani & Bloom, 2021).

**Fig. 5:**
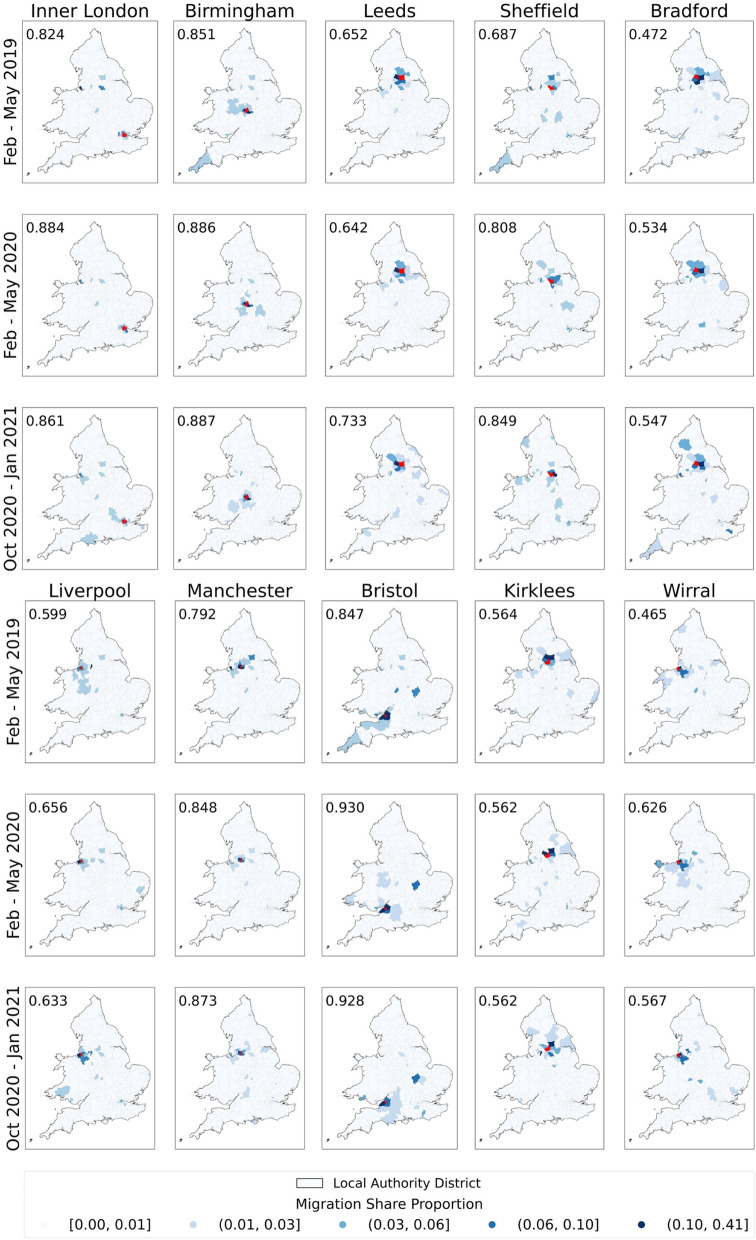
Destination LADs for those who moved out of the top 10 most populous cities in England and Wales before the pandemic, during the first and second city-exiting waves. The selected city is marked in red. Other locations are coloured in blue shades by their migration share from the selected city. The proportion of moving to LADs other than large cities is marked on the top left corner of each map

Restrictive policies have had uneven impacts on cities in England and Wales with the largest population sizes. Figure 5 illustrates the home locations of people who moved out of those cities before the pandemic (February to May 2019), during the first wave (February to May 2020) and the second wave (October 2020 to January 2021) of migration out of cities. In the figure, the origin city is marked in red, and other LADs are coloured in blue by their share of the migrating population. The 13 LADs in Inner London except for the City of London were counted as Inner London, whereas for the other cities, only LADs located in the city centre were counted. Based on the migration share, we calculated the proportion of moving to LADs other than large cities, which is marked on the top left corner of each map. The average of this proportion in the three periods was 0.675, 0.738 and 0.744, respectively. Regardless of the epidemic, the majority of those who moved out of the cities chose to go to the nearby countryside, while this trend is more pronounced during the pandemic.

Liverpool and Wirral had a lower migration share to rural areas, due to a large flow between these two conjunctive cities. The same pattern was also shown at Leeds, Bradford, and Kirklees. The average migration share to rural areas from these five cities in the three periods were 0.550, 0.604 and 0.608, while the average of the other cities were 0.800, 0.871 and 0.880. Therefore, if there is a conjunctive large city, people would have a similar willingness to move to the conjunctive city and rural areas, but if there is not, most moved to rural areas. During the pandemic, more people moved to rural areas regardless of whether there is a conjunctive city or not.

The majority of those moving out of the cities chose to go to somewhere nearby. However, a small number of people showed a particular interest in specific areas that were not necessarily near their home locations in the original city. For example, those exiting from Bristol showed a keen interest in moving to North Northamptonshire (9.6% and 8.2% in the first and second period, respectively), and those from Inner London areas tended to move to Liverpool (3.8% and 4.8%). Before the pandemic, Cornwall, a holiday destination in the southwest corner of the UK, attracted a large number of residents from Birmingham, Sheffield and Bristol. During the pandemic, although people’s destination choices were more dispersed than before it, fewer people moved to holiday destinations.

**Fig. 6:**
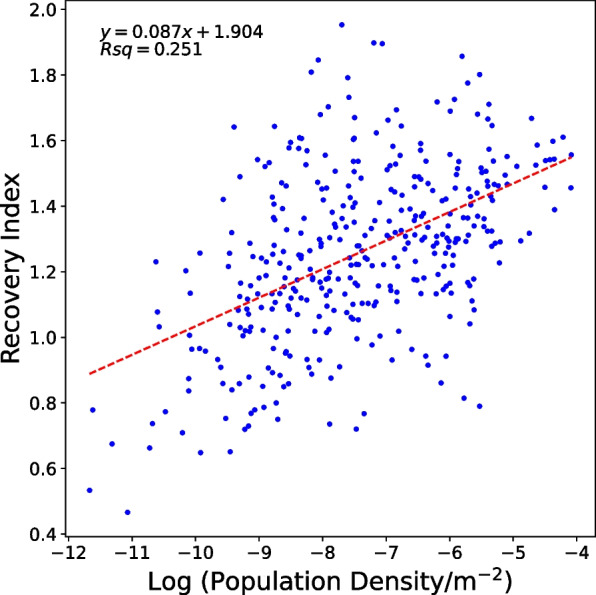
The plot of the recovery index against (log) population density

The recovery index reflects how quickly each LAD recovered in 2021 relative to its status in 2020 in terms of migration. Here, we used the recovery index to evaluate population recovery after the three waves in the number of COVID-19 cases and lockdowns. Of the 370 LADs analysed, 311 (84%) had a recovery index of more than 1, meaning that most parts of the UK experienced more internal migrations and hence recovered. Additionally, when the index was plotted against population density (Fig. 6), the slope of the trendline was greater than 0, meaning that LADs with higher population density (i.e., large cities) recovered faster. This was represented by a significantly higher increase in internal migrations, both moving in and out.

## Conclusion and future work

Fine-grained, near real-time and economic migration monitoring will undoubtedly contribute to a better understanding of human mobility and migration behaviour. This study developed a data-driven workflow for extracting migration matrices from Twitter data and demonstrated its applications through a case study of the UK before and during the COVID-19 pandemic. Through LAD-scale estimation of monthly migration, we illustrated the potential of Twitter data in human mobility studies. The observed trends in migration clearly reflect the impacts of pandemic measures, and our LAD-scale results are consistent with yearly internal migration flow data from the UK government. Notably, in contrast to our monthly results obtained from Twitter data, ONS migration data are updated annually with a delay of more than 6 months and cover England and Wales only. Our results and the method developed here can help in evaluating the effectiveness of policies enacted in response to the rapidly changing pandemic and also help guide the local and national post-pandemic recovery.

By presenting five migration indicators, our analysis revealed a decline in migration during the pandemic and highlights the spatial heterogeneity of migration in the UK. Namely, residents moved out of large cities during the pandemic, a pattern that was significant during the first and second lockdowns. These patterns are consistent with other studies conducted in other countries and regions using other types of data (Tønnessen, 2021; Willberg et al., 2021). We also found that if there was a conjunctive city, residences of large cities had a similar willingness to move there and to rural areas; otherwise, it was mainly to rural areas. It was more likely to move to rural areas during the pandemic. Moreover, the number of people moving into cities after the lockdowns are far less than those moving out of cities during lockdowns, despite the faster recovery in large cities.

This study has some potential limitations in terms of the data source and the methodology for data processing, some of which could be improved in future works. As the Twitter Developer API only exposes about 1% of all tweets, and the proportion of Twitter users varies across LADs, limitations in the samples make migration patterns difficult to identify and less reliable. Also, the population sample bias in Twitter data may leave some social groups underrepresented (Sloan et al., 2015). By comparing with ONS migration statistics, we have proved that accumulated Twitter data is useful at the LAD scale for monthly migration monitoring. Comparisons with data from other sources, such as other social networks or mobile apps, could further improve the effectiveness and accuracy of the results. In addition, this work implemented a simple method of estimating home locations on a monthly basis. The identified locations might not be home locations, but regular activity places. Although the method used here has been proven useful for trend analysis, an improved approach may increase the accuracy or update frequency. Besides, due to the temporal sparseness of Twitter data, this study generated aggregated location-based OD matrixes. By using continuously tracked data, such as mobile phone data, individual OD matrixes could be generated for geographical flow analysis.

Despite the limitations, our work potentially contributes to migration monitoring in other social events. In addition to the pandemic, the used process is crucial in other fast-changing social events, such as refugee migration across Europe during the Ukraine war (Juric, 2022), tens of billions of population migration during Chunyun – the Spring Festival travel rush in China (Xiao et al., 2021), and the flocking of the population after the Haiti earthquake (Lu et al., 2012). Moreover, we made our code open sourced and generated monthly migration matrix. Using the Twitter developer API and our code, updated data sets can be generated as well. The methods can be easily applied to other similar or integrated or enhanced data sets.

## Data Availability

Some of the data that supports the findings of this work were derived from the following resources available in the public domain: https://geoportal.statistics.gov.uk, https://coronavirus.data.gov.uk/. Due to restrictions in the Twitter terms of service (https://twitter.com/en/tos) and the Twitter developer policy (https://developer.twitter.com/en/developer-terms/agreement-and-policy.html), we cannot provide the raw tweets used in this study. Twitter explicitly forbids the transfer of tweets between third parties. Nevertheless, we have produced minimal research data, including coding for processing the same type of data and the generated migration index, for another researcher to verify the results.
